# Supplementary material for: Long-Term Outcomes after Non-Traumatic Out-of-Hospital Cardiac Arrest in Pediatric Patients: A Systematic Review
Source: J Clin Med. 2022 Aug 26;11(17):5003. doi: 10.3390/jcm11175003 (PMC9457161; doi:10.3390/jcm11175003)
Supplement: Supplementary file 1 [file jcm-11-05003-s001.zip › Supplementary Material S1.pdf]

Supplementary Material S1. Full Search Phrases Used for the Three Respective Databases

6 Oct

Medline - 581 papers found

1. OHCA.tw. or exp Out-of-Hospital Cardiac Arrest/
2. ("out of hospital" or out-of-hospital).ab,ti.
3. Exp Heart Arrest/ OR ((heart or cardiac or cardiopulmonary or circulat* or ventricular or sudden) adj3 (arrest or death or standstill or asystole*)).ab,ti
4. 2 AND 3
5. 1 OR 4
6. exp Mortality/ or exp Outcome Assessment, Health Care/ or exp Patient Reported Outcome Measures/ or Exp Quality of Life/ OR ((long-term or longterm) and (outcome* or effect or effects or mortalit* or death* or survival* or survivor* or prognosis or quality of life or QOL or health utilities index or SF-36 or ED-5D or EQ-5D-5L or anxiety or PTSD or post-traumatic-stress-disorder or depression or mental health or psychological or neuropsychological or function or functional outcome or participation)).tw.
7. 5 AND 6
8. (exp Newborn/ OR exp Infant/ OR exp Pediatrics/ OR exp Child/ OR exp Teenager/ OR exp Adolescent/ OR exp Youth/) OR (Neonat* or Newborn* or Infant* or Pediatric* or Paediatric* or Peds or Bab* or Child* or Adolescen* or Preteen* or Pre-teen* or Teen* or Youth* or Minor*).ab,ti
9. 7 AND 8

1	OHCA.tw. or exp Out-of-Hospital Cardiac Arrest/	6415	Advanced	Display Results More
2	("out of hospital" or out-of-hospital).ab,ti.	11866	Advanced	Display Results More
3	exp Heart Arrest/ or ((heart or cardiac or cardiopulmonary or circulat* or ventricular or sudden) adj3 (arrest or death or standstill or asystole*)).ab,ti.	129423	Advanced	Display Results More
4	2 and 3	8043	Advanced	Display Results More
5	1 or 4	9631	Advanced	Display Results More
6	exp Mortality/ or exp Outcome Assessment, Health Care/ or exp Patient Reported Outcome Measures/ or exp Quality of Life/ or ((long-term or longterm) and (outcome* or effect or effects or mortalit* or death* or survival* or survivor* or prognosis or quality of life or QOL or health utilities index or SF-36 or ED-5D or EQ-5D-5L or anxiety or PTSD or post-traumatic-stress-disorder or depression or mental health or psychological or neuropsychological or function or functional outcome or participation)).tw.	2163608	Advanced	Display Results More
7	5 and 6	3388	Advanced	Display Results More
8	exp Newborn/ or exp Infant/ or exp Pediatrics/ or exp Child/ or exp Teenager/ or exp Adolescent/ or exp Youth/ or (Neonat* or Newborn* or Infant* or Pediatric* or Paediatric* or Peds or Bab* or Child* or Adolescen* or Preteen* or Pre-teen* or Teen* or Youth* or Minor*).ab,ti.	4726265	Advanced	Display Results More
9	7 and 8	581	Advanced	Display Results More

Embase - 73 papers found

1. 'OHCA'/de or 'Out-of-Hospital Cardiac Arrest'/exp
2. ('out of hospital' OR 'out-of-hospital'):ti,ab
3. 'Heart Arrest'/exp OR ((heart OR cardiac OR cardiopulmonary OR circulat* OR ventricular OR sudden) NEAR/3 (arrest OR death OR standstill OR asystole*)):ti,ab
4. 2 AND 3
5. 1 OR 4
6. 'Mortality'/exp OR exp 'Outcome Assessment, Health Care'/exp OR 'Patient Reported Outcome Measures'/exp OR 'Quality of Life'/exp OR (('long-term' or 'longterm') AND (outcome* OR effect OR effects OR mortalit* OR death* OR survival* OR survivor* OR prognosis or quality of life OR QOL OR health utilities index OR SF-36 OR ED-5D OR EQ-5D-5L OR anxiety OR PTSD OR post-traumatic-stress-disorder OR depression OR mental health OR psychological OR neuropsychological OR function OR functional outcome OR participation)):ti,ab
7. 5 AND 6
8. ('Newborn'/exp OR 'Infant'/exp OR 'Pediatrics'/exp OR 'Child'/exp OR 'Teenager'/exp OR 'Adolescent'/exp OR 'Youth'/exp) OR (Neonat* or Newborn* or Infant* or Pediatric* or Paediatric* or Peds or Bab* or Child* or Adolescen* or Preteen* or Pre-teen* or Teen* or Youth* or Minor*):ti,ab
9. 7 AND 8

#9	#7 AND #8	73
#8	'newborn'/exp OR 'infant'/exp OR 'pediatrics'/exp OR 'child'/exp OR 'teenager'/exp OR 'adolescent'/exp OR 'youth'/exp OR neonat*:ti,ab OR newborn*:ti,ab OR infant*:ti,ab OR pediatric*:ti,ab OR paediatric*:ti,ab OR peds:ti,ab OR bab*:ti,ab OR child*:ti,ab OR adolescen*:ti,ab OR preteen*:ti,ab OR 'pre teen*':ti,ab OR teen*:ti,ab OR youth*:ti,ab OR minor*:ti,ab	5,289,754
#7	#5 AND #6	792
#6	('mortality'/exp OR exp) AND 'outcome assessment, health care'/exp OR 'patient reported outcome measures'/exp OR 'quality of life'/exp OR (('long-term':ti,ab OR 'longterm':ti,ab) AND (((((outcome*:ti,ab OR effect:ti,ab OR effects:ti,ab OR mortalit*:ti,ab OR death*:ti,ab OR survival*:ti,ab OR survivor*:ti,ab OR prognosis:ti,ab OR quality:ti,ab) AND of:ti,ab AND life:ti,ab OR qol:ti,ab OR health:ti,ab) AND utilities:ti,ab AND index:ti,ab OR 'sf 36':ti,ab OR 'ed 5d':ti,ab OR 'eq 5d 5l':ti,ab OR anxiety:ti,ab OR ptsd:ti,ab OR 'post traumatic stress disorder':ti,ab OR depression:ti,ab OR mental:ti,ab) AND health:ti,ab OR psychological:ti,ab OR neuropsychological:ti,ab OR function:ti,ab OR functional:ti,ab) AND outcome:ti,ab OR participation:ti,ab))	706,765
#5	#1 OR #4	15,970
#4	#2 AND #3	13,635
#3	'heart arrest'/exp OR (((heart OR cardiac OR cardiopulmonary OR circulat* OR ventricular OR sudden) NEAR/3 (arrest OR death OR standstill OR asystole*)):ti,ab)	214,484
#2	'out of hospital':ti,ab OR 'out-of-hospital':ti,ab	18,566
#1	'ohca'/de OR 'out-of-hospital cardiac arrest'/exp	11,632

Cochrane - 68 papers found

1. OHCA:ti,ab
2. MeSH descriptor: [Out-of-Hospital Cardiac Arrest] explode all trees
3. #1 OR #2
4. (Out-of-Hospital):ti,ab
5. (Out of Hospital):ti,ab
6. #4 OR #5
7. MeSH descriptor: [Heart Arrest] explode all trees
8. ((heart or cardiac or cardiopulmonary or circulat* or ventricular or sudden) NEAR/3 (arrest or death or standstill or asystole*)):ti,ab
9. #7 OR #8
10. #6 AND #9
11. #3 OR #10
12. MeSH descriptor: [Mortality] explode all trees
13. MeSH descriptor: [Outcome Assessment, Health Care] explode all trees
14. MeSH descriptor: [Patient Reported Outcome Measures] explode all trees
15. MeSH descriptor: [Quality of Life] explode all trees
16. ("long-term" or "long term"):ti,ab
17. (outcome* or effect or effects or mortalit* or death* or survival* or survivor* or prognosis or "quality of life" or QOL or "health utilities index" or "SF-36" or "ED-5D" or "EQ-5D-5L" or anxiety or PTSD or "post-traumatic-stress-disorder" or depression or "mental health" or psychological or neuropsychological or function or "functional outcome" or participation):ti,ab
18. #16 AND #17
19. #12 OR #13 OR #14 OR #15 OR #18
20. #11 AND #19
21. (Neonat* or Newborn* or Infant* or Pediatric* or Paediatric* or Peds or Bab* or Child* or Adolescen* or Preteen* or Pre-teen* or Teen* or Youth* or Minor*):ti,ab
22. MeSH descriptor: [Infant, Newborn] explode all trees
23. MeSH descriptor: [Pediatrics] explode all trees
24. MeSH descriptor: [Child] explode all trees
25. MeSH descriptor: [Adolescent] explode all trees
26. #21 OR #22 OR #23 OR #24 OR #25
27. #20 AND #26

#1	OHCA:ti,ab	Limits	481
#2	MeSH descriptor: [Out-of-Hospital Cardiac Arrest] explode all trees	MeSH	459
#3	#1 OR #2	Limits	761
#4	(Out-of-Hospital):ti,ab	Limits	1790
#5	(Out of Hospital):ti,ab	Limits	16360
#6	#4 OR #5	Limits	16360
#7	MeSH descriptor: [Heart Arrest] explode all trees	MeSH	2060
#8	((heart or cardiac or cardiopulmonary or circulat* or ventricular or sudden) NEAR/3 (arrest or death or standstill or asystole*)):ti,ab	Limits	10812
#9	#7 OR #8	Limits	11305
#10	#6 AND #9	Limits	1448
#11	#3 OR #10	Limits	1561
#12	MeSH descriptor: [Mortality] explode all trees	MeSH	13665
#13	MeSH descriptor: [Outcome Assessment, Health Care] explode all trees	MeSH	154482
#14	MeSH descriptor: [Patient Reported Outcome Measures] explode all trees	MeSH	813
#15	MeSH descriptor: [Quality of Life] explode all trees	MeSH	26469
#16	("long-term" or "long term"):ti,ab	Limits	101143
#17	(outcome* or effect or effects or mortalit* or death* or survival* or survivor* or prognosis or "quality of life" or QOL or "health utilities index" or "SF-36" or "ED-5D" or "EQ-5D-5L" or anxiety or PTSD or "post-traumatic-stress-disorder" or depression or "mental health" or psychological or neuropsychological or function or "functional outcome" or participation):ti,ab	Limits	1184223
#18	#16 AND #17	Limits	82637
#19	#12 OR #13 OR #14 OR #15 OR #18	Limits	243217
#20	#11 AND #19	Limits	379
#21	(Neonat* or Newborn* or Infant* or Pediatric* or Paediatric* or Peds or Bab* or Child* or Adolescen* or Preteen* or Pre-teen* or Teen* or Youth* or Minor*):ti,ab	Limits	229580
#22	MeSH descriptor: [Infant, Newborn] explode all trees	MeSH	16781
#23	MeSH descriptor: [Pediatrics] explode all trees	MeSH	709
#24	MeSH descriptor: [Child] explode all trees	MeSH	58731
#25	MeSH descriptor: [Adolescent] explode all trees	MeSH	107314
#26	#21 OR #22 OR #23 OR #24 OR #25	Limits	313798
#27	#20 AND #26	Limits	68
