# Supplementary material for: Long-Term Outcomes after Non-Traumatic Out-of-Hospital Cardiac Arrest in Pediatric Patients: A Systematic Review
Source: J Clin Med. 2022 Aug 26;11(17):5003. doi: 10.3390/jcm11175003 (PMC9457161; doi:10.3390/jcm11175003)
Supplement: Supplementary file 1 [file jcm-11-05003-s001.zip › Supplementary Material S2.pdf]

Supplementary Material S2: Characteristics of Included Studies and Patients with Out-of-Hospital Cardiac Arrest

**Author**	**Year**	**Study Year Range**	**Country**	**Region**	**Type of Study**	**Sample size**	**Age (mean SD)**	**Gender (Male)**	**Risk of Bias (NOS)**
Albrecht et al. [25]	2021	2002-2019	Rotterdam, Netherlands	Europe	Cohort study	360	3.4 [median]	225	Selection - 4<br>Comparability - 1<br>Outcome - 3<br>Total - 8
Dieckmann et al. [22]	1995	1989-1993	San Francisco, USA	North America	Retrospective cohort study	65	1.51 (NA)	42	Selection - 4<br>Comparability - 1<br>Outcome - 3<br>Total - 8
Hickson et al. [24]	2021	2008-2018	USA	North America	Retrospective cohort study	17	2 (1.62)	10	Selection - 4<br>Comparability - 1<br>Outcome - 2<br>Total - 7
Hunfeld et al. *[26]*	2021	2012-2017	Netherlands	Europe	Prospective case control study	113	6.42 (9.48)	33	Selection - 4<br>Comparability - 1<br>Outcome - 3<br>Total - 8

Lee et al. [28]	2019	2011-2015	Taiwan	Asia	Retrospective population study	2178	NA	NA	Selection - 4<br>Comparability - 1<br>Outcome - 3<br>Total - 8
Silka et al. [23]	2017	2004-2016	Los Angeles, USA	North America	Retrospective case series analysis	45	4 (NA)	45	Selection - 4<br>Comparability - 2<br>Outcome - 3<br>Total - 9
Suominen et al. [27]	2014	1987-2007	Helsinki, Finland	Europe	Prospective long term follow up	21	3.23 (2.94)	12	Selection - 3<br>Comparability - 1<br>Outcome - 3<br>Total - 7
